# What is the Relationship between Functional Limitations, Pain, and Self-Perceptions of Aging?

**DOI:** 10.1093/geroni/igab046.2319

**Published:** 2021-12-17

**Authors:** Jillian Minahan Zucchetto, Ashley Blasi

**Affiliations:** 1 Fordham University, United States; 2 Fordham University, Bronx, New York, United States

## Abstract

Self-perceptions of aging (SPA) influences health and mortality during older adulthood (e.g., Kotter-Grühn et al., 2009; Sargent-Cox et al., 2012). Westerhof and Wurm (2015) found that increasing functional limitations (FL) worsened older adults’ SPA. Additional research is needed to identify other factors that influence SPA. Although pain is common among older adults and is a frequent cause of disability (e.g., Brooks et al., 2019), it has not been examined as a factor influencing SPA. Pain is often misperceived as an inevitable part of aging because of widely held negative stereotypes about aging (Thielke et al., 2012). The experience of pain may activate internalized negative stereotypes about aging, which may worsen SPA. Thus, this study investigated: 1) the relationship between chronic and recent pain, FL, and SPA, and 2) the interactive effect of FL and pain on SPA within a sample of community-dwelling adults aged 65 years and older. This study included 5,126 participants from the 2014 wave of the Health and Retirement Study. Controlling for covariates, chronic pain (β = .09, p < .001) and recent pain (β = .12, p < .001) were associated with negative SPA and were stronger than FL (β = .04, p < .01). There was also a small interaction between FL and recent pain on SPA (β = -.03, p < .01) such that the negative impact of FL on SPA was stronger among individuals who reported low pain. These findings highlight the importance of pain in older adults’ evaluation of their own aging.

